# Effect of core stability exercises and Russian electrical stimulation on nonspecific low back pain: a single-blinded randomized controlled trial

**DOI:** 10.1038/s41598-025-28313-x

**Published:** 2025-12-17

**Authors:** Noura Nayel, Hesham Ezzat, Sabreen Ahmed, Haitham Saleh

**Affiliations:** 1 Department of Physical Therapy for Basic Science, Faculty of Physical Therapy, Deraya University, Minya, Egypt; 2 Department of Human Anatomy, Faculty of Medicine, Deraya University, Minya, Egypt; 3 Head of Basic Science Department, Faculty of Physical Therapy, Innovation University, Cairo, Egypt

**Keywords:** Low back pain, Russian electrical stimulation therapy, Core stability exercises, Muscle strength, Randomized controlled trial, Musculoskeletal system, Anatomy, Rheumatology, Signs and symptoms

## Abstract

Nonspecific low back pain (NSLBP) is a predominant contributor to disability worldwide. Core stability exercises (CSEs) serve as a common intervention; however, their therapeutic effects may be augmented through the incorporation of adjunct modalities such as neuromuscular electrical stimulation. This randomized controlled trial aimed to evaluate whether the addition of Russian current (RC) stimulation to a CSE regimen enhances improvements in pain, functional capacity, muscle thickness, and spinal stability among patients diagnosed with NSLBP compared with a regimen comprising CSE alone. This single-blinded, randomized controlled trial was conducted at the outpatient clinic of Deraya University over a six-week intervention period. A total of fifty patients aged between 20 and 25 years with chronic NSLBP were recruited. The participants were randomly allocated into two groups via a closed-envelope. Group 1 (the study group, n = 25) received CSE and RC three times a week for six weeks, whereas Group 2 (the control group, n = 25) received CSE only. The primary outcomes assessed included pain intensity, measured via the visual analog scale (VAS), and functional disability, evaluated via the Oswestry Disability Index (ODI). The secondary outcomes included the muscle thicknesses of the transversus abdominis (TrA) and lumbar multifidus (LM), which were measured via ultrasonography, and lumbar spine stability, which was assessed via a Spinal Mouse device. The data were analyzed via mixed-model MANOVA for group and time effects, accompanied by post hoc Bonferroni correction. Between-group comparisons were performed via independent t tests, whereas within-group analyses were performed via paired t tests. There was a significant decrease in the VAS score and ODI and a significant increase in the stability score, TrA, and LM thickness, especially in group 1. The demographic characteristics of the groups were comparable at baseline (p > 0.05). Compared with Group 2 (CSE alone), Group 1 (CSE + RC) exhibited significantly greater improvements in the VAS score (MD: -1.04, 95% CI: -1.79–0.29, p = 0.008), ODI (MD: -9.76%, 95% CI: -12.13–7.39, p < 0.001), stability score (MD: 7.53%, 95% CI: 0.95–14.12, p = 0.02), and muscle thickness (e.g., right TrA: MD: 0.08 cm, 95% CI: 0.02–0.12, p = 0.004). There was a significant decrease in the VAS score and ODI and an increase in the stability score, TrA, and LM thickness, especially in group 1. Compared with core stability exercises alone, the Russian current accompanied by core stability exercises results in greater decreases in pain and disability and greater increases in functional ability, TrA and LM thickness, and lumbar spine stability.

**Trial registration: **The study was registered on clinicaltrials.gov (NCT06495099) on 02/07/2024.

**Supplementary Information:**

The online version contains supplementary material available at 10.1038/s41598-025-28313-x.

## Introduction

Low back pain (LBP) is a highly prevalent musculoskeletal disorder that affects approximately 80% of individuals at some point in their lives^1^. It is the second leading cause of work absences and a primary reason for seeking healthcare services^2^.

LBP is typically classified as nonspecific (90%) or specific (10%) on the basis of whether a discernible pathoanatomical cause can be established^3^.

The symptoms may be categorized according to their duration: acute (lasting less than six weeks), subacute (six to twelve weeks), or chronic (exceeding twelve weeks)^4^. It is estimated that chronic low back pain (CLBP) afflicts approximately 50% of the general population, and nearly 70% of adults experience at least one episode of LBP during their lifetime^5^.

Recent epidemiological studies indicate a concerning increase in the incidence of nonspecific low back pain (NSLBP) among young and middle-aged individuals^6^. This trend is particularly alarming for university students, as research elucidates a heightened vulnerability due to prolonged screen exposure and insufficient physical activity within their daily routines^7^. Furthermore, suboptimal sitting postures may escalate mechanical stress on the lumbar spine, amplifying static loads on the lumbar joint ligaments^8^. Prolonged sitting can predispose individuals to diminished lumbar lordosis and an increase in posterior pelvic tilt, subsequently leading to discomfort or pain^9^.

Approximately 85% of all LBP cases are classified as nonspecific, indicative of conditions not involving inflammation, anatomical alterations, or specific underlying pathologies^10^. Nonspecific LBP is frequently correlated with abnormalities in neuromuscular function, restricted flexibility of the lumbar muscles, and decreased spinal mobility^11^.

Nonspecific low back pain (NSLBP) is a prevalent condition influenced by several physical and psychological risk factors, including prolonged standing or ambulation, heavy lifting, poor postural habits, and sedentary behavior. Additional risk factors contributing to the onset of NSLBP include obesity and lifestyle choices characterized by nutritional imbalance^12^.

Research indicates that patients suffering from chronic NSLBP frequently exhibit weakness and delayed activation of deep stabilizing muscles, specifically the lumbar multifidus (LM) and transversus abdominis (TrA), which can culminate in postural instability and spinal dysfunction^2^.

The contemporary management of NSLBP is multifactorial, integrating pharmacological interventions (e.g., non-steroidal anti-inflammatory drugs [NSAIDs]), non-pharmacological strategies (e.g., patient education and reassurance), and physical therapy modalities. Among these, exercise therapy focused on core stabilization has emerged as a cornerstone intervention endorsed by international guidelines owing to its efficacy in enhancing both pain relief and functional capacity. Notably, the role of neuromuscular electrical stimulation (NMES), particularly in conjunction with exercise for muscle strengthening, has received increasing attention in recent studies^13^.

Core stabilization exercise (CSE), also referred to as specific stabilization or motor control exercise, is frequently employed as a therapeutic intervention aimed at addressing lower back pain^14^. The primary objective of CSE is to enhance neuromuscular control and strengthen the local trunk muscles, specifically the LM and TrA, to facilitate the restoration of their roles in maintaining segmental spinal stability. However, a prominent clinical challenge persists; many patients encounter difficulties in voluntarily isolating and activating these inhibited stabilizers, thereby limiting the overall effectiveness of exercise therapy alone^15^.

Electrotherapy is widely recognized for its analgesic properties and its ability to mitigate inflammation, enhance the range of motion, and contribute to muscle strengthening^16^. For example, the Russian current (RC), characterized by its asymmetrical sinusoidal or bipolar current train with a frequency of 2500 Hz, is modulated in bursts at 50 Hz. This type of electrical stimulation has the potential to induce involuntary, targeted muscle contractions. Notably, this modality aids in the activation of deep stabilizing muscles that are typically challenging to engage consciously^17^.

Despite the individual benefits associated with both CSE and Russian electrical stimulation, high-quality randomized controlled trials (RCTs) that rigorously examine the effects of Russian current on low back pain (LBP) are lacking. Furthermore, the synergistic effects of combining CSE with RES on outcomes related to NSLBP have not been thoroughly investigated. Few studies have employed objective assessment measures, such as ultrasound imaging and spinal mouse stability scores, to evaluate these outcomes effectively.

The present study seeks to bridge this research gap by systematically assessing the impact of augmenting core stability exercises with Russian current on various outcomes, including pain reduction, quality of life, the morphological thickness of the TrA and LM muscles, and the stability of the lumbar spine in individuals diagnosed with nonspecific low back pain.

### Null hypothesis

There will be no significant difference in pain, disability, muscle thickness, or lumbar stability between patients with nonspecific low back pain (NSLBP) who receive core stability exercises combined with Russian electrical stimulation and those who receive core stability exercises alone.

### Alternate hypothesis (H₁)

Patients with nonspecific low back pain (NSLBP) who receive core stability exercises combined with Russian electrical stimulation show significantly greater improvements in pain, disability, muscle thickness, and lumbar stability than those who receive core stability exercises alone.

## Materials and methods

### Study design

This single-blinded randomized controlled trial, in which participants were unaware of their assigned treatment group, was conducted at the outpatient clinic of the Faculty of Physical Therapy at Deraya University. The assessor responsible for all outcome measurements, including the visual analog scale (VAS), Oswestry Disability Index (ODI), ultrasound assessments, and Spinal Mouse evaluations, maintained blinding concerning group allocation throughout the study. Randomization of participants was achieved by categorizing patients with odd identification numbers into the treatment group, whereas those with even identification numbers were assigned to the control group. The physical therapist responsible for administering the interventions, which included both CSE and RC, was unable to maintain blinding due to the inherent nature of the electrotherapy application. This process involves tangible aspects such as equipment setup, parameter adjustments, and observable muscle contractions. To mitigate potential bias in the study, a highly standardized treatment protocol was implemented. Furthermore, the statistician conducting the data analysis was blinded to the group assignments, and the independent reviewer was not involved in the application of the interventions, thereby enhancing the integrity of the findings. The present study was meticulously designed, executed, and reported in accordance with the CONSORT 2010 statement, thereby ensuring adherence to principles of transparency and completeness in clinical trial reporting. This prospective randomized controlled trial received prior approval from the ethical committees of the Faculty of Physical Therapy at Deraya University, documented under approval number DCSR-02024-06. Furthermore, it is registered with ClinicalTrials.gov under the identifier NCT06495099 as of 09/07/2024. The trial was first registered on 02/7/2024, and all procedures followed the appropriate guidelines and regulations.

**The inclusion criteria were as follows **NSLBP patients aged 20–25 years who had experienced symptoms for more than 3 months and whose BMI ranged from 18.5 to 24.9. With pain ratings above four on the visual analog scale (VAS). An age-related disability score of 19% or higher was given by the Oswestry Disability Questionnaire.

**The exclusion criteria** included neurological or musculoskeletal illnesses impacting the lumbar spine; pregnancy and lactation; a history of lumbar spine surgery; indications of lumbar radiculopathy or myelopathy; and indications of severe pathology, such as malignancy, inflammatory disorders, or infection. Participants who had engaged in any structured exercise or physical therapy program for low back pain within the past three months, those utilizing systemic corticosteroids, and those who routinely consumed analgesics or nonsteroidal anti-inflammatory drugs (NSAIDs) for low back pain more than twice a week or muscle relaxants in the preceding four weeks were also excluded. Finally, individuals exhibiting cognitive impairments that could hinder their ability to follow instructions, as well as those with a recent history of trauma (within the last six months) to the back or pelvis, were disqualified from participation.

### Sample size

A sample size calculation was conducted via G. Power (version 3.1.9.7), and the findings indicated that each group needed a sample size of 25 people. The computations were performed with a significance level of α = 0.05 and a statistical power of 80%, and an effect size of 0.88 was calculated based on a previous pilot study and is considered large according to Cohen’s conventions^17^.

### Participants

From a pool of 58 individuals with nonspecific low back pain (NSLBP), fifty patients were selected from the outpatient clinic of Deraya University in Minya. Patients were diagnosed with chronic nonspecific low back pain, classified under the International Classification of Diseases, Eleventh Revision (ICD-11), specifically designated as code MG30.0 for chronic primary low back pain. The initial diagnosis was conducted by a board-certified orthopedic specialist with more than ten years of clinical experience. Following this assessment, the validity of the diagnosis was confirmed by the lead researcher in physical therapy to determine eligibility for inclusion in the study. Patients meeting the specified inclusion criteria were referred by their treating physicians, and participation in the study was formalized through the signing of informed consent documents by those who agreed to participate.

**Fig. 1:**
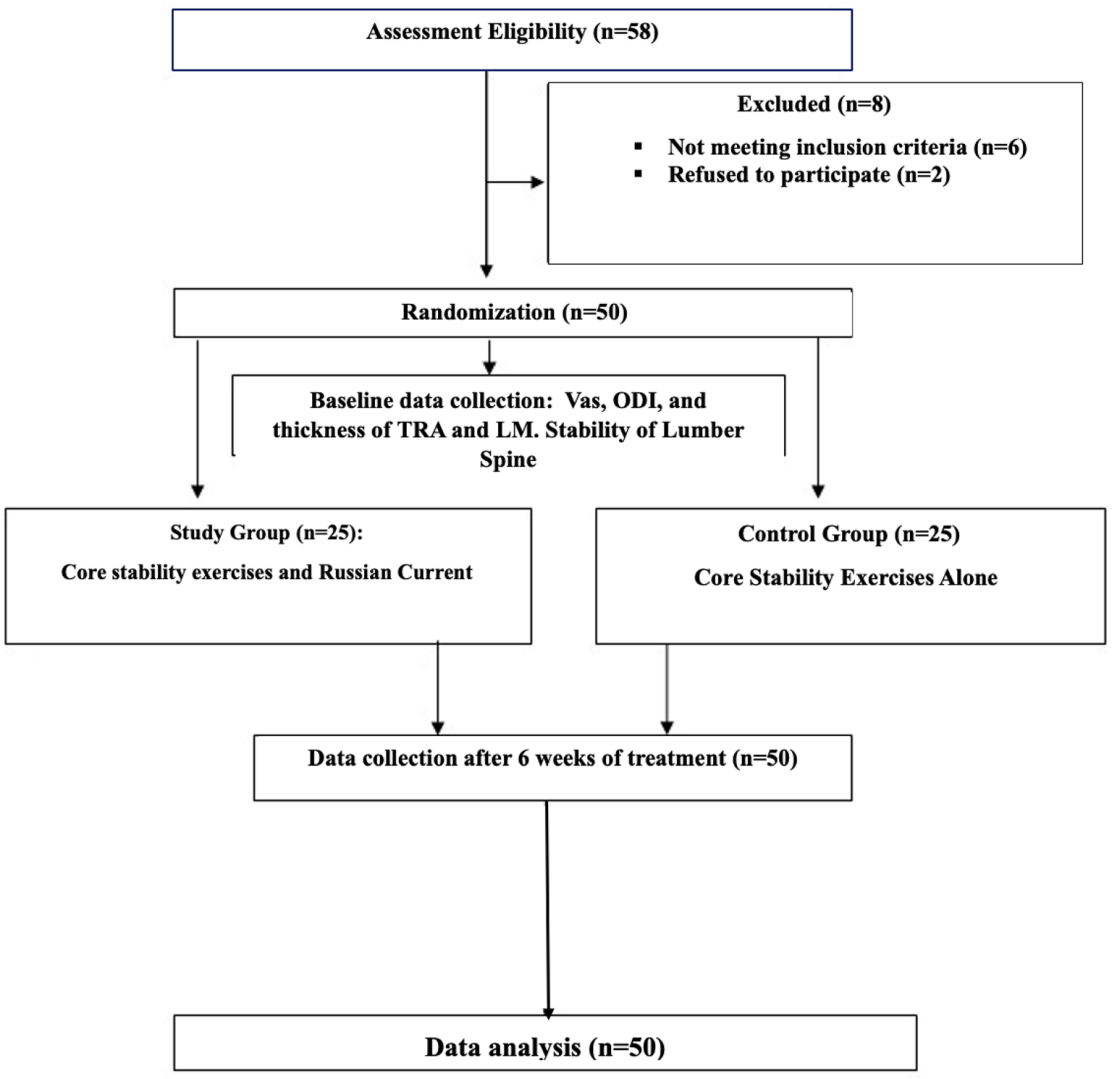
Flow chart of randomization.

The subjects were randomly assigned to two groups via the closed envelope method (see Fig. 1). Group 1 (study group, *n* = 25) was given Russian electrical stimulation accompanied by core stability exercises three times a week for six weeks. Group 2 (control group, *n* = 25) was given core stability exercises three times a week for six consecutive weeks, and reassessment of the patients was performed after the last session of the planned treatment period. Both groups were assessed by the VAS score, the ODI, ultrasonography, and the use of a spinal mouse device before and after treatment.

### Assessment tools

The visual analog scale (VAS) was used to assess pain levels. The VAS is a reliable and valid tool for measuring low back pain (LBP) and consists of a 10 cm line where a score of 0 indicates no pain and a score of 10 represents the most severe pain imaginable. The visual analog scale (VAS) is a reliable and valid instrument for evaluating pain^18^.

Functional disability was evaluated via the Oswestry Disability Index (ODI), a well-established and valid tool for assessing functional impairment in patients with low back pain. The index values range from 0 to 100% and are categorized for analysis: scores between 0 and 19% signify minimal disability, 20–39% denote moderate disability, 40–59% reflect severe disability, 60–79% represent crippling disability, and scores from 80 to 100% indicate bedridden individuals^19^.

**Fig. 2:**
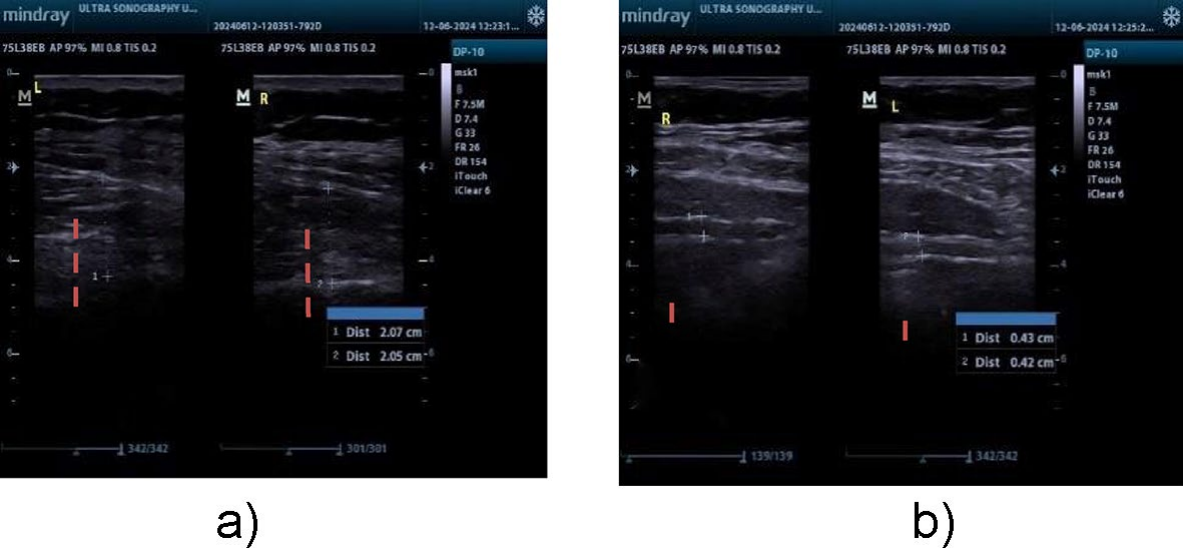
(**a**) LM thickness measurement, (**b**) TRA thickness measurement.

Ultrasonography is a dependable and valid technique with strong intrarater reliability for assessing transverse abdominis and lumbar multifidus muscle thickness in patients with chronic low back pain (CLBP). All measurements of muscle thickness in this research were conducted by one operator, guaranteeing strong interrater reliability. Research has consistently demonstrated elevated ICCs for thickness assessments and contraction ratios in the transversus abdominis and lumbar multifidus muscles under both static (ICC = 0.71–0.99) and semidynamic conditions (ICC = 0.73–0.98)^20^. Ultrasonography was utilized to assess the LM and TrA muscles. Mindray DP10 ultrasonography was used. For the LM muscle, participants were positioned face down with a cushion under the abdomen to reduce lumbar lordosis. The thickness was measured between the fascial plane separating the muscle from the subcutaneous tissue and the posterior aspect of the L4-5 facet joint^21^ (Fig. 2a).

r the TrA muscle, a linear ultrasonic transducer was placed horizontally on the abdominal wall at the midpoint between the iliac crest and the lower edge of the rib cage, with its inner edge approximately 10 cm from the midline. The individual was positioned comfortably on their back, with a cushion positioned beneath the knees. The probe was placed with its inner edge approximately 10 cm away from the midline. The probe’s ultimate placement was adjusted to guarantee that the medial edge of the transversus abdominis (TrA) was approximately 2 cm away from the medial edge of the ultrasound image while the participant was in a relaxed position^21^ (Fig. 2b).

The stability and mobility of the lumbar spine were characterized via a surface-based Spinal Mouse system.

The Spinal Mouse system (IDIAG M360©, Fehraltdorf, Switzerland) is a reliable and credible tool for evaluating spinal curvatures, flexibility, and alignment, with high intraclass correlation coefficients (ICCs) ranging from 0.86 to 0.98^22^. The device was positioned paravertebrally along the spinous processes from C7 to S3 to capture the contour of the skin over the vertebral bodies. All outcome measures were assessed at baseline and after the six-week intervention^23^. Conversely, motion capture systems provide enhanced features for in-depth biomechanical assessment, focusing on kinematic and kinetic measurements, with ICC values surpassing 0.90^24^. Moreover, the isokinetic dynamometer is noted for its outstanding reliability in measuring muscle strength, with ICC values ranging from 0.93 to 0.99^25^.

All outcome measures were assessed at two separate time points: at baseline, before starting the first intervention session, and immediately after completing the 6-week intervention program.

### Intervention procedures

**Fig. 3:**
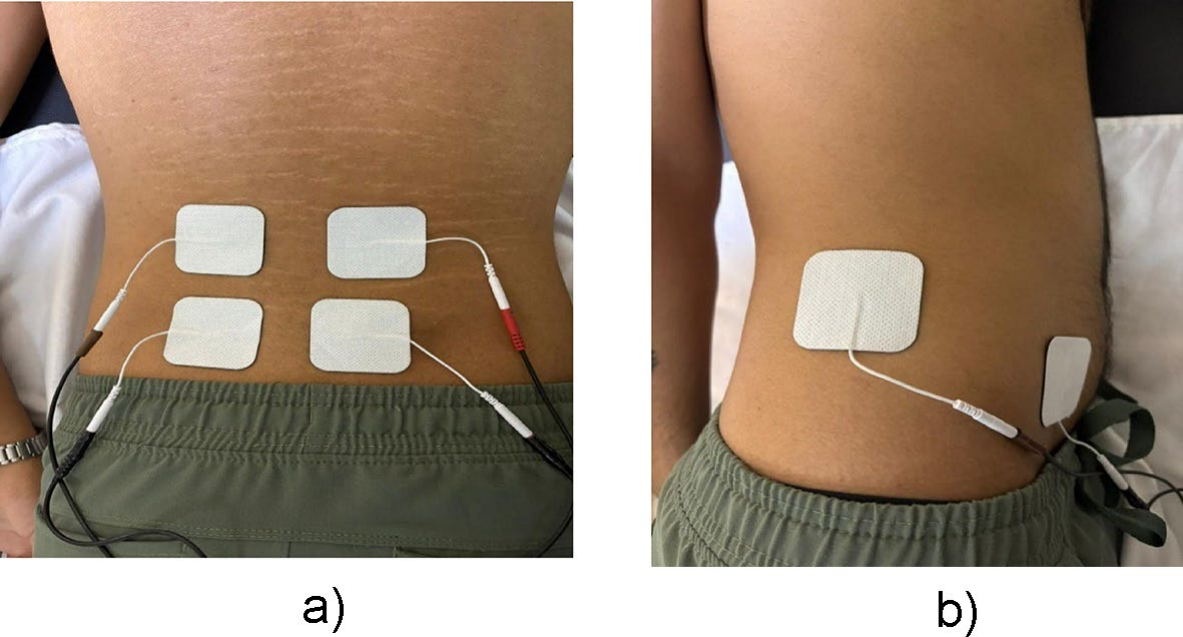
(**a**) Electrode placement for LM, (**b**) Electrode placement for TRA.

Russian electrical stimulation (RES) was applied three times a week for six weeks. The RES was delivered via an ITO ES-5200 electrotherapy device with a frequency of 2500 Hz, a 50 Hz burst modulation, and a 50% duty cycle. The stimulation strength varied between 31 and 58 mA. First, the amplitude was established at a minimal level adequate for a full and prolonged isometric contraction of the lumbar paraspinal muscles. The amplitude is then gradually elevated to the individual’s maximum tolerance to enhance muscle activation^26^. Participants were positioned in a prone posture to facilitate targeted activation of the lumbar multifidus (LM) muscle. Electrodes were strategically placed bilaterally on the L4 and L5 spinous processes, ensuring optimal conduction of electrical stimulation during the intervention (Fig. 3a). For the transversus abdominis (TrA) muscle, a reference electrode was placed on the mid-axillary line 1 cm above the iliac crest. The active electrode was positioned 2 cm above and 2 cm medial to the anterior superior iliac spine (Fig. 3b)^21^. The treatment protocol followed the 10/50/10 method: a 10-second muscle contraction followed by 50 s of rest, repeated 10 times, for a total duration of 20 min^17^.

Core stabilization exercises were included in the intervention for both groups as the standard of care for nonspecific low back pain (NSLBP), thereby allowing the study to isolate the specific additive effect of Russian electrical stimulation. These exercises are a form of motor control training focused on activating and controlling deep spinal muscles, particularly the transversus abdominis (TrA) and lumbar multifidus (LM).

The capacity for TrA activation was assessed via a Stabilizer Pressure Biofeedback Unit (PBU) (Chattanooga Group, Australia). The participants were positioned in a prone posture on a firm surface, with the PBU placed over the TrA muscles, just superior to the anterior superior iliac spines. The PBU was inflated to a pressure of 70 mmHg. The participants were instructed to gently draw their lower abdomen away from the sensor without moving their back or hips, aiming for a pressure decrease of 4–10 mmHg. This contraction was held for 10 s and repeated 10 times^2^.

Lumbar multifidus activation was facilitated with participants in a crook-lying position. The PBU was maintained at 40 mmHg to monitor spinal control. The participants were instructed to take a relaxed breath, keep their chest still, and attempt to draw their sacrum toward their shoulders, which resulted in a pressure drop of 5–10 mmHg.

**Fig. 4:**
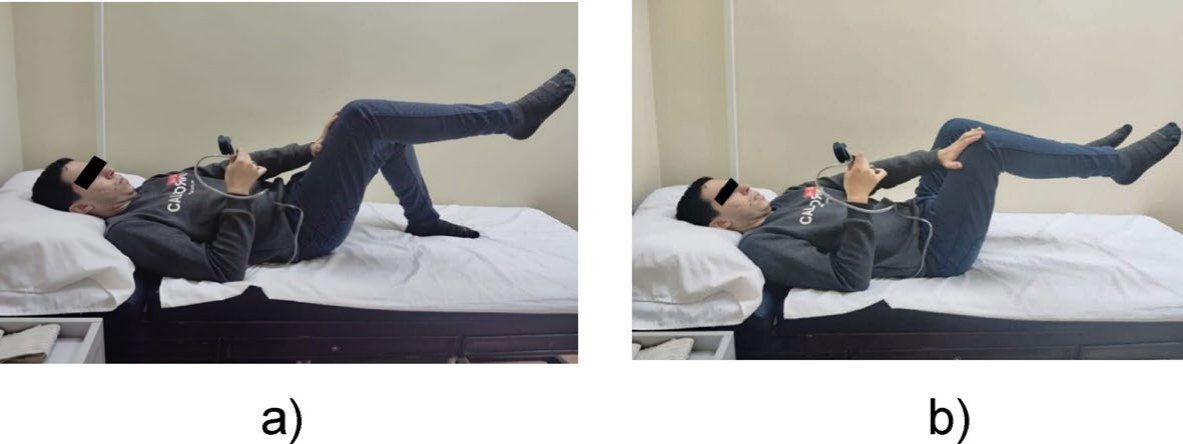
**(a)** Isometric opposite knee-to-hand push, (**b**) Isometric knee**-**to**-**hand push + 2nd heel lift.

The exercise protocol progressed according to the participant’s ability to activate the muscles while maintaining spinal control. Exercises included:

1. Static diagonal: Isometric opposite knee-to-hand push: This method starts by promoting activation of the lumbar multifidus via a pressure biofeedback unit (PBU) adjusted to 30–35 mmHg or by using one’s hands to maintain consistent pressure, resulting in spinal control. Slowly raise one knee toward the opposite hand, pressing it isometrically against each other along a diagonal path. This pressure was maintained for 10 s, and the procedure was repeated 10 times, guaranteeing consistency (no fluctuation in pressure). Should there be any rise or fall in recorded pressure, the movement should be stopped immediately, and the patient should return to the initial position. (Fig. 4a)
2. Static diagonal heel lift: The same isometric knee-to-hand press was used with the addition of elevating the second heel off the ground and holding the position for 10 s, and the procedure was repeated 10 times (Fig. 4b).
3. Alternate Single Leg Heel Touch: While in a crook-lying position with the hips flexed to 90 degrees, the participants alternately lowered one heel to the ground^27^.

Exercises progressed in complexity from stable positions (e.g., crook-lying) to dynamic movements as participants gained control. It was emphasized that all exercise should be pain free, as discomfort could indicate incorrect forms or excessive intensity^27^.

### Statistical analysis

All the data were analyzed via the Statistical Package for the Social Sciences (SPSS) version 25 for Windows (IBM, Chicago, IL, USA). The statistical significance threshold for all tests was set at *p* < 0.05.

An unpaired t test was used to perform a comparative analysis of baseline subject characteristics between the groups. A chi-square analysis was conducted to compare the sex distributions. The Shapiro–Wilk test was used to assess the statistical normality of the data, whereas Levene’s test was used to evaluate the homogeneity of variances between the groups. A mixed MANOVA was employed to examine the effects of the intervention on the primary outcome variables, including the visual analog scale (VAS) score, Oswestry Disability Index (ODI), stability score, and thickness of the transversus abdominis (TrA) and lumbar multifidus (LM) muscles. To account for multiple comparisons, post hoc analyses were performed via the Bonferroni correction. Management of Variables and Uniformity: Possible confounding factors such as age, sex, BMI, and initial outcome scores were managed via the randomized assignment procedure.

## Results

### Subject characteristics

Table 1 displays the means ± SDs of the participants’ demographic attributes, such as age, height, weight, and BMI. During the study, retention was total, with no participants missing or dropping out from any group. Given the lack of dropouts, an intention-to-treat analysis was conducted, including data from all 50 participants who were randomized in the study. There was no notable difference between the groups regarding age, weight, height, BMI, or sex distribution (*p* > 0.05). Treatment compliance and adherence: This research revealed a strong adherence rate, as participants in both experimental categories attended more than 90% of the planned sessions (18 sessions) throughout a six-week period. Importantly, no major negative effects were observed during the treatment phase. Nonetheless, two people in the current Russian group noted mild, temporary erythema localized beneath the electrodes. This condition resolved on its own and did not require any pause in the treatment process.

Table 1 shows the subject characteristics of groups 1 and 2.

**Table 1:** Comparison of subject characteristics between group 1 and group 2.

	Group 1	Group 2	*p* value
	Mean ± SD	Mean ± SD	
Age (in years)	22.64 ± 1.87	22.24 ± 1.94	0.46
Weight (in kilogram)	66.51 ± 11.16	68.53 ± 10.01	0.50
Height (cm)	165.84 ± 6.36	167.64 ± 7.91	0.38
BMI (kg/m²)	24.11 ± 3.42	24.27 ± 2.27	0.84
Sex, N (%)			
Females	15 (60%)	12 (48%)	0.39
Males	10 (40%)	13 (52%)	

SD, standard deviation; p value, probability value.

#### Effects of treatment on the VAS score, ODI, stability score, TrA, and multifidus thickness

A mixed MANOVA revealed that therapy and time had a significant interaction effect (F = 66.16, *p* = 0.001, ηp² = 0.92). A statistically significant main effect of treatment was observed (F = 4.34, *p* = 0.001; ηp²= 0.42). The main effect of time was statistically significant (F = 268.69, *p* = 0.001; ηp**²**= 0.0.98).

### Within-group comparison

Significant improvements were observed in both groups when posttreatment scores were compared with baseline measurements across all outcome variables. A notable decrease in the visual analog scale (VAS) score and the Oswestry Disability Index (ODI) score was evident, alongside a considerable increase in the stability score for both groups (*p* > 0.001).

**Group 1**, which received the combined therapy, demonstrated a mean decrease of 4.4 in the VAS score and a 23.68% decrease in the ODI score. These improvements surpassed the **minimum clinically important difference (MCID)** standards of 2.0 points for the VAS score and 10 points for the ODI, indicating that the observed enhancements were both statistically significant and clinically relevant. The average percentage changes in the VAS, ODI, and stability scores for this group were 63.22%, 72.11%, and 23.91%, respectively. In **Group 2**, which received only core stability exercises, the corresponding percentage changes were 48.86% for the VAS score, 43.69% for the ODI, and 12.83% for the stability score, as illustrated in Table 2.

With respect to muscle thickness, considerable posttreatment increases were noted in the right and left transversus abdominis (TrA) and multifidus muscles in both groups compared with their respective pretreatment measurements (*p* > 0.001). For **Group 1**, the thicknesses of the right and left TrA and multifidus muscles increased by 37.50%, 27.27%, 22.77%, and 24.75%, respectively. In contrast, **Group 2** had smaller percentage increases of 9.09%, 9.37%, 6.90%, and 3.85%, respectively (Table 2).

### Between-group comparison

Before the intervention, no statistically significant difference was found between the groups (*p* > 0.05). However, posttreatment comparisons revealed a significant difference in favor of Group 1, with greater improvements in the VAS score, ODI, stability score, and thickness of the TrA and multifidus muscles (*p* < 0.05). The **partial eta-squared (ηp²)** values obtained from the MANOVA are detailed in the tables for further examination of effect sizes (Tables 2 and 3).

**Table 2:** Mean VAS score, ODI score, and stability score before and after treatment in groups 1 and 2:.

	Pre treatment	Post treatment	MD	95% CI	% of change	*p* value
	Mean ± SD	Mean ± SD				
VAS						
Group 1	6.96 ± 1.21	2.56 ± 1.26	4.4	3.79: 5.01	63.22	0.001
Group 2	7.04 ± 1.02	3.60 ± 1.38	3.44	2.83: 4.05	48.86	0.001
MD	−0.08	−1.04				
95% CI	−0.72: 0.56	−1.79: −0.29				
	*p* = 0.80	*p* = 0.008				
ηπ^2^		0.14				
ODI (%)						
Group 1	32.84 ± 9.95	9.16 ± 4.33	23.68	20.13: 27.23	72.11	0.001
Group 2	33.60 ± 7.82	18.92 ± 4.01	14.68	11.13: 18.23	43.69	0.001
MD	−0.76	−9.76				
95% CI	−5.85: 4.33	−12.13: −7.39				
	*p* = 0.77	*p* = 0.001				
ηπ^2^		***0.59***				
Stability score (%)						
Group 1	45.46 ± 11.70	56.33 ± 10.52	−10.87	−13.04: −8.69	23.91	0.001
Group 2	43.25 ± 10.94	48.80 ± 12.55	−5.55	−7.72: −3.37	12.83	0.001
MD	2.21	7.53				
95% CI	−4.23: 8.65	0.95: 14.12				
	*p* = 0.49	*p* = 0.02				
ηπ^2^		***0.1***				

SD, standard deviation; MD, mean difference; CI, confidence interval; ηp^2,^ partial eta-squared^,^ p value, probability value.

**Table 3:** Mean TrA and multifidus thicknesses before and after treatment in groups 1 and 2:.

	Pre treatment	Post treatment	MD	95% CI	% of change	*p* value
	Mean ± SD	Mean ± SD				
Right TrA thickness (cm.)						
Group 1	0.32 ± 0.08	0.44 ± 0.07	−0.12	−0.13: −0.10	37.5	0.001
Group 2	0.33 ± 0.09	0.36 ± 0.10	−0.03	−0.05: −0.02	9.09	0.001
MD	−0.01	0.08				
95% CI	−0.06: 0.04	0.02: 0.12				
	*p* = 0.73	*p* = 0.004				
ηπ^2^		***0.16***				
Left TrA thickness (cm.)						
Group 1	0.33 ± 0.07	0.42 ± 0.09	−0.09	−0.11: −0.08	27.27	0.001
Group 2	0.32 ± 0.09	0.35 ± 0.10	−0.03	−0.05: −0.02	9.37	0.001
MD	0.01	0.07				
95% CI	−0.04: 0.05	0.02: 0.12				
	*p* = 0.76	*p* = 0.01				
ηπ^2^		***0.13***				
Right multifidus thickness (cm.)						
Group 1	2.02 ± 0.41	2.48 ± 0.43	−0.46	−0.51: −0.42	22.77	0.001
Group 2	2.03 ± 0.52	2.17 ± 0.55	−0.14	−0.18: −0.10	6.9	0.001
MD	−0.01	0.31				
95% CI	−0.28: 0.25	0.03: 0.59				
	*p* = 0.93	*p* = 0.03				
ηπ^2^		***0.1***				
Left multifidus thickness (cm.)						
Group 1	1.98 ± 0.43	2.047 ± 0.47	−0.49	−0.54: −0.45	24.75	0.001
Group 2	2.08 ± 0.56	2.16 ± 0.58	−0.08	−0.13: −0.03	3.85	0.001
MD	−0.1	0.31				
95% CI	−0.38: 0.19	0.02: 0.62				
	*p* = 0.49	*p* = 0.03				
ηπ^2^		***0.09***				

SD, standard deviation; MD, mean difference; CI, confidence interval; ηp^2^^,^ partial eta-squared^;^ p value, probability value.

## Discussion

This study was designed to investigate the effects of a combined intervention consisting of core stability exercises and Russian electrical stimulation on pain, quality of life, lumbar stability, and the thickness of the transversus abdominis (TrA) and lumbar multifidus (LM) muscles in individuals with nonspecific low back pain.

The results revealed a significant reduction in both the visual analog scale (VAS) score and the Oswestry Disability Index (ODI) score in both treatment groups compared with their respective pretreatment values (*p* > 0.001). Additionally, notable increases in the stability score and thickness of the TrA and LM muscles were observed.

A posttreatment intersubject-group comparison revealed statistically significant differences, with the combined therapy group showing greater improvements in VAS and ODI scores, along with a more substantial increase in both the stability score and the thickness of the TrA and LM muscles (*p* < 0.001). The observed decreases in the VAS and ODI scores surpassed the minimum clinically important difference, confirming that the changes were not only statistically significant but also clinically meaningful for patients.

While studies specifically on the effect of Russian current on lower back pain (LBP) are limited, electrotherapy in general has demonstrated efficacy in pain management. This effect is often explained by the gate-control theory proposed by Melzack and Wall. This theory posits that the transmission of noxious stimuli through small-diameter, unmyelinated C fibers and myelinated A-delta fibers can be inhibited by the activation of large-diameter, nonnociceptive A-beta fibers. The activation of these A-beta fibers stimulates inhibitory interneurons in the spinal cord, which in turn “close the gate” to painful stimuli, preventing their transmission to the brain^28^. When used in appropriate settings, the Russian current selectively activates these A-beta fibers. Consequently, increased A-beta activity reduces the perception of pain. Other proposed mechanisms for the pain-relieving effects of transcutaneous electrical nerve stimulation (TENS) include the activation of descending inhibitory pathways within the central nervous system through the release of endogenous opioids^29^ or a reduction in proinflammatory cytokines such as IL-1 and IL-6 in the bloodstream^30^.

Electrotherapeutic methods, which use transdermal electric currents^31^, are commonly used by physiotherapists because of their beneficial effects on muscle performance and high patient acceptance^32–34^. Neuromuscular electrical stimulation (NMES) activates nerve fibers in a specific sequence. It begins with the activation of Aβ fibers, which produce the sensation of paresthesia that marks the sensory threshold. As the current amplitude increases, the NMES may also activate C fibers, which transmit pain signals. If the current exceeds the motor threshold, muscle contractions may occur^35^. The findings of this study align with existing research on the benefits of electrotherapy. For example, a randomized trial by Batistella et al. revealed that, compared with a control group, a group receiving current Russian therapy presented significant and sustained reductions in both the VAS score and the Oswestry low back disability index^17^. Similarly, Lee et al. (2017) reported significant improvements in muscle resistance, thickness, and pain levels following current Russian therapy for quadriceps pain after ACL reconstruction^36^.

To further support the efficacy of the Russian current, Çankaya et al. demonstrated its added benefits in managing symptoms and improving functional mobility and quality of life for individuals with patellofemoral pain^37^. Rajan et al. noted a slight advantage of current Russian therapy over other treatments, such as interferential therapy and conventional exercise, for short-term pain reduction and muscle strength improvement in patients with postoperative lower limb fractures^38^. Among the various NMES modalities designed for muscle strengthening, medium-frequency currents such as Aussie (AC) and Russian (RC**)** currents are particularly notable for their effectiveness.

Low-frequency currents, such as functional electrical stimulation (FES), are known to increase motor unit recruitment, which contributes to increased muscle strength and pain relief^39,40^. Specifically, the Russian current can trigger the depolarization of sensory and motor nerve fibers, stimulate fast Type II motor units and thereby promote muscle contractions that lead to an increase in muscle strength^41^. This finding is supported by studies such as Heggannavar et al., who demonstrated that Russian electrical stimulation effectively improved quadriceps strength and functional capacity in patients with primary osteoarthritis (OA) knee pain^42^. Similarly, Janarthanan et al. reported that the integration of Russian current and resistance exercises resulted in increased pain relief, strength, and performance in sprinters with calf muscle strain^43^. Further research by Letícia Cittadin et al. revealed a significant increase in muscle thickness in the nondominant hand of healthy females^39^, and a review by Pereira et al. suggested that the Russian current was the most effective method for promoting strength and thickness gains^33^.

However, some studies present conflicting findings. For example, Prabha et al. reported no additional benefits from the Russian current when supervised exercises were already being performed for primary knee osteoarthritis^28^.

For individuals with chronic nonspecific low back pain (NSLBP), a common issue is the delayed activation of deep stabilizing muscles, which leads to postural instability and exacerbated pain and dysfunction^44^. This study revealed a 23.91% increase in stability scores in the experimental group compared with a 12.83% increase in the control group, suggesting that Russian electrical stimulation (RES) promotes the recruitment of these weakened stabilizers^41^. Nevertheless, a known drawback of core stability exercises is the challenge in engaging these deep stabilizing muscles due to neuromuscular inhibition^45^. Furthermore, a study by Naka et al. on patients with chronic neck and lower back pain revealed that while suprathreshold electrotherapy administered six times per week improved lumbar flexibility, it did not significantly alter pain or disability perceptions^46^.

The integration of neuromuscular electrical stimulation (NMES), particularly with methodologies such as Russian current, alongside physical exercise, offers several unique benefits. This combined approach can help reduce neuromuscular inhibition, which in turn facilitates earlier and more effective activation of weakened stabilizing muscles, such as the transversus abdominis (TrA) and lumbar multifidus (LM). These muscles are often difficult for patients to activate voluntarily, leading to functional impairments. Consequently, this combined method has the potential to accelerate gains in muscle strength and promote structural changes. However, there are important considerations and potential drawbacks associated with this integrated approach. These include the risk of muscle fatigue, the possibility of skin irritation beneath the electrodes, and the reliance on specialized equipment and clinical expertise for proper implementation. These factors may limit the accessibility and practicality of this treatment for certain patient populations and healthcare settings^41^.

The findings of this research have significant clinical and academic implications. For clinicians, the evidence supporting the efficacy of combined therapy (CSE + RC) provides a practical framework for achieving faster and more pronounced results in pain relief, functional enhancement, and spinal stabilization. For patients, this protocol offers a more efficient path to recovery, potentially mitigating the long-term effects associated with chronic pain. Furthermore, for the research community, this study contributes to the current understanding of the NMES and establishes a validated framework for future investigations. This could facilitate additional research into the long-term effects and comparative effectiveness of this method against other electrotherapy modalities.

## Conclusion

This study revealed that a six-week intervention combining Russian electrical stimulation with core stability exercises is significantly more effective than core stability exercises alone in reducing pain and disability in young adults with chronic nonspecific low back pain. The integrated program also led to a significant increase in the thickness of both the transversus abdominis and lumbar multifidus muscles, along with improvements in lumbar stability. These results support the incorporation of this combined treatment method into clinical practice for this patient population, highlighting its potential to enhance patient outcomes in the management of chronic low back pain.

### Limitations and recommendations

This study has several limitations that warrant further discussion for future research. First, the study design did not include long-term follow-up assessments, which limits the understanding of whether the effects of the Russian current (RC) on neuromuscular changes are sustained over time. Future research should incorporate follow-up evaluations at 3 to 6 months to address this limitation. Second, the study was conducted on a sample of young adults, which limits the generalizability of the findings to other populations, such as older adults or those with significant spinal pathologies. It is recommended that future studies evaluate the impact of these rehabilitation interventions across a broader range of demographics, including older adults, athletes, and individuals with different body mass index (BMI) classifications. Third, this study focused on the additive effect of RC on core stability exercises. Future research should compare the effectiveness of RC with that of other electrotherapy methods, such as transcutaneous electrical nerve stimulation (TENS), to determine its relative efficacy. Fourth, while the results generally revealed medium to large effect sizes, certain comparisons, particularly for left multifidus thickness, revealed smaller effect sizes (ηp² = 0.09) and wider confidence intervals. This finding indicates that while the intervention was effective overall, the magnitude of the additional benefit from the Russian current varied significantly among individuals for this specific outcome. Fifth, in addition to the methodological limitations, the use of a single blinded evaluator and therapist, while essential for feasibility, introduces a potential for bias that could influence the results. Additionally, the strict inclusion criteria, although helpful in creating a uniform sample, also limit the direct applicability of the findings to a broader patient population. Sixth, a sham electrotherapy control group was not included. This prevents the definitive exclusion of a placebo effect influencing the results observed in the current Russian group.

Furthermore, a single-blinded design, while necessary for the feasibility of the intervention, presents a potential drawback. Although the participants and the outcome evaluators were blinded, the treating therapist could not be due to the nature of applying the Russian current. Despite implementing standardized procedures for interactions and obscuring the data analyst’s identity, the risk of performance bias cannot be eliminated. Future research should prioritize the development of a robust sham electrotherapy protocol to enable a double-blind design, which would strengthen the validity of the findings by minimizing potential biases.

## Supplementary Information

Below is the link to the electronic supplementary material.

Supplementary Material 1

## Data Availability

The datasets used and analyzed during the current study are available from the corresponding author upon reasonable request.

## Abbreviations

CNSLBP: Chronic nonspecific low back pain
CSE: Core stabilization exercise
RES: Russian electrical stimulation
VAS: Visual analog scale
ODI: Oswestry Disability Index
TrA: Transversus abdominis muscle
LM: Lumbar multifidus muscle
CI: Confidence interval
