# Supplementary material for: Effect of core stability exercises and Russian electrical stimulation on nonspecific low back pain: a single-blinded randomized controlled trial
Source: Sci Rep. 2025 Dec 17;15:44053. doi: 10.1038/s41598-025-28313-x (PMC12715220; doi:10.1038/s41598-025-28313-x)
Supplement: Supplementary file 1 — Supplementary Material 1 [file 41598_2025_28313_MOESM1_ESM.pdf]

	group	sex	VASpre	VASpost	ODIpre	ODIpost	stabilitypr	stabilitypo	RTtrapre	RTtrapost	Lttrapre	LTtrapost	rtlmpre	rtlmpost	ltlmpre	ltlmpost	age	weight	height	bmi
1	1	M	9.00	4.00	50.00	18.00	52.00	68.30	0.43	0.48	0.42	0.52	2.05	2.57	2.07	2.51	25.00	73.20	168.00	25.94
2	1	M	8.00	2.00	54.00	16.00	50.00	60.00	0.43	0.51	0.37	0.45	1.43	1.97	1.51	2.12	23.00	71.70	165.00	26.34
3	1	M	7.00	3.00	40.00	18.00	38.60	60.00	0.36	0.50	0.40	0.57	2.79	3.22	2.77	3.32	21.00	77.20	169.00	27.03
4	1	F	6.00	0.00	20.00	5.00	26.30	45.00	0.31	0.41	0.24	0.31	1.83	2.24	1.84	2.33	20.00	87.40	172.00	29.54
5	1	F	8.00	4.00	28.00	8.00	43.60	50.00	0.42	0.53	0.39	0.50	2.36	2.75	2.29	2.62	22.00	89.50	165.00	32.87
6	1	F	6.00	2.00	22.00	18.00	33.60	36.30	0.45	0.50	0.31	0.38	2.59	3.41	2.64	3.05	24.00	80.20	171.00	27.43
7	1	F	8.00	3.00	26.00	5.00	42.00	57.30	0.25	0.32	0.20	0.27	2.01	2.44	1.67	2.11	25.00	75.50	164.00	28.07
8	1	F	8.00	3.00	44.00	12.00	44.00	55.00	0.37	0.45	0.29	0.38	2.07	2.52	1.88	2.33	20.00	66.10	155.00	27.51
9	1	F	6.00	0.00	26.00	7.00	46.30	62.50	0.30	0.39	0.30	0.42	1.74	2.14	1.76	2.36	20.00	58.40	163.00	21.98
10	1	F	8.00	3.00	42.00	12.00	67.60	67.70	0.19	0.32	0.21	0.32	1.86	2.24	1.89	2.45	22.00	64.20	169.00	22.48
11	1	M	8.00	3.00	22.00	7.00	56.60	57.00	0.37	0.44	0.38	0.43	1.93	2.45	1.97	2.51	23.00	66.10	163.00	24.88
12	1	M	6.00	4.00	29.00	6.00	58.60	65.30	0.33	0.43	0.38	0.51	1.22	1.66	1.10	1.66	24.00	70.30	168.00	24.91
13	1	F	8.00	5.00	30.00	7.00	44.00	65.00	0.29	0.37	0.35	0.44	1.51	2.02	1.30	1.92	25.00	55.50	166.00	20.14
14	1	F	8.00	4.00	40.00	8.00	25.60	46.00	0.39	0.51	0.35	0.45	2.14	2.66	2.02	2.31	24.00	61.40	170.00	21.25
15	1	M	6.00	2.00	49.00	10.00	51.00	59.00	0.36	0.47	0.43	0.53	2.06	2.58	2.08	2.38	25.00	65.20	173.00	21.78
16	1	F	5.00	2.00	29.00	7.00	25.30	35.50	0.36	0.50	0.38	0.46	1.54	1.98	1.52	1.85	25.00	76.20	175.00	24.88
17	1	F	7.00	3.00	27.00	9.00	42.60	49.00	0.29	0.49	0.41	0.58	2.80	3.23	2.88	3.80	24.00	64.20	168.00	22.75
18	1	M	5.00	1.00	31.00	10.00	32.60	36.50	0.24	0.40	0.25	0.32	1.84	2.25	1.85	2.33	24.00	73.90	171.00	25.27
19	1	M	7.00	2.00	25.00	3.00	41.00	56.30	0.35	0.52	0.40	0.51	2.37	2.76	2.30	2.73	22.00	65.40	179.00	20.41
20	1	F	8.00	2.00	43.00	9.00	43.00	49.60	0.38	0.49	0.32	0.39	2.66	3.12	2.55	3.06	21.00	53.30	155.00	22.19
21	1	F	5.00	1.00	25.00	5.00	45.50	61.50	0.18	0.31	0.21	0.28	2.11	2.45	1.68	2.31	20.00	60.70	165.00	22.30
22	1	F	7.00	2.00	41.00	8.00	60.60	66.70	0.30	0.44	0.30	0.39	2.08	2.53	2.22	2.69	21.00	59.80	160.00	23.36
23	1	M	7.00	2.00	21.00	10.00	55.60	65.50	0.23	0.38	0.31	0.43	1.75	2.15	1.76	1.97	20.00	49.20	157.00	19.96
24	1	F	5.00	3.00	28.00	5.00	67.60	74.30	0.12	0.31	0.22	0.33	1.87	2.25	2.04	2.55	22.00	46.00	155.00	19.15
25	1	M	8.00	4.00	29.00	6.00	43.00	59.00	0.30	0.43	0.39	0.44	1.84	2.46	1.95	2.65	24.00	52.20	160.00	20.39
1	2	M	6.00	6.00	22.00	20.00	56.00	72.00	0.31	0.34	0.31	0.32	1.23	1.27	1.23	1.29	25.00	65.20	158.00	26.12
2	2	M	5.00	5.00	24.00	24.00	61.60	66.00	0.37	0.40	0.38	0.39	1.66	1.69	1.81	1.95	20.00	59.80	155.00	24.89
3	2	M	7.00	3.00	42.00	25.00	41.00	53.00	0.42	0.44	0.42	0.47	1.95	2.19	1.69	1.72	25.00	47.60	154.00	20.07
4	2	F	8.00	6.00	28.00	22.00	50.60	53.00	0.21	0.25	0.21	0.23	2.24	2.36	2.10	2.16	23.00	48.90	155.00	20.35
5	2	F	9.00	3.00	38.00	16.00	27.00	28.00	0.24	0.29	0.23	0.28	1.96	2.20	2.02	2.11	21.00	55.80	160.00	21.80
6	2	F	9.00	4.00	36.00	20.00	44.60	48.00	0.24	0.27	0.25	0.24	2.03	2.14	2.22	2.29	20.00	67.80	160.00	26.48
7	2	F	7.00	5.00	40.00	18.00	31.00	38.00	0.25	0.29	0.22	0.27	2.53	2.58	2.55	2.90	22.00	56.40	160.00	22.03
8	2	F	8.00	5.00	45.00	15.00	41.00	43.00	0.51	0.55	0.39	0.53	3.26	3.43	3.16	3.22	24.00	57.80	163.00	21.75
9	2	F	6.00	5.00	21.00	19.00	55.00	64.00	0.32	0.35	0.33	0.34	1.24	1.28	1.24	1.28	25.00	62.10	165.00	22.81
10	2	M	7.00	4.00	23.00	23.00	60.60	65.00	0.38	0.41	0.40	0.41	1.67	1.77	1.82	1.86	20.00	66.20	168.00	23.46
11	2	M	6.00	2.00	41.00	31.00	40.00	52.00	0.43	0.45	0.44	0.49	1.96	2.20	1.74	1.77	20.00	69.00	170.00	23.88
12	2	F	7.00	5.00	27.00	21.00	49.60	52.00	0.22	0.26	0.29	0.25	2.25	2.37	2.11	2.17	22.00	72.20	173.00	24.12
13	2	F	8.00	2.00	37.00	15.00	26.00	27.00	0.25	0.30	0.25	0.30	1.97	2.01	2.03	2.06	23.00	70.50	175.00	23.02
14	2	M	8.00	3.00	35.00	15.00	43.60	47.00	0.25	0.28	0.22	0.25	2.04	2.15	2.43	2.49	24.00	72.40	178.00	22.85
15	2	M	6.00	4.00	39.00	19.00	30.00	38.00	0.26	0.30	0.23	0.28	2.54	2.59	2.71	2.91	25.00	67.70	172.00	22.88
16	2	F	7.00	4.00	39.00	19.00	40.00	42.00	0.52	0.56	0.50	0.52	3.17	3.44	2.27	2.31	21.00	68.90	159.00	27.25
17	2	M	7.00	4.00	30.00	18.00	54.00	60.00	0.33	0.36	0.34	0.35	1.29	1.32	1.25	1.33	20.00	82.50	174.00	27.25
18	2	M	6.00	3.00	22.00	21.00	53.50	58.00	0.39	0.42	0.41	0.42	1.68	1.95	1.83	1.87	25.00	83.50	171.00	28.56
19	2	F	8.00	1.00	40.00	18.00	39.00	51.00	0.44	0.46	0.45	0.50	1.97	2.25	1.91	1.98	24.00	77.40	175.00	25.27
20	2	M	8.00	4.00	26.00	15.00	48.60	51.00	0.23	0.27	0.24	0.26	2.26	2.38	2.12	2.18	23.00	82.50	171.00	28.21
21	2	M	7.00	1.00	36.00	14.00	25.00	26.00	0.26	0.31	0.26	0.31	1.98	2.02	2.04	2.22	22.00	72.50	174.00	23.95
22	2	F	7.00	2.00	34.00	13.00	42.60	46.00	0.26	0.29	0.23	0.27	2.15	2.22	2.25	2.30	21.00	73.90	172.00	24.98
23	2	M	6.00	3.00	38.00	17.00	29.00	36.00	0.27	0.31	0.24	0.29	2.15	2.60	2.82	2.92	20.00	77.80	175.00	25.40
24	2	M	6.00	3.00	48.00	18.00	39.00	41.00	0.53	0.57	0.44	0.53	2.29	2.45	3.38	3.41	21.00	79.30	176.00	25.60
25	2	F	7.00	3.00	29.00	17.00	53.00	63.00	0.34	0.37	0.35	0.36	1.26	1.41	1.26	1.29	20.00	75.50	178.00	23.83
